# Current Nanomedicine for Targeted Vascular Disease Treatment: Trends and Perspectives

**DOI:** 10.3390/ijms232012397

**Published:** 2022-10-17

**Authors:** Kyung-A Choi, June Hyun Kim, Kitae Ryu, Neha Kaushik

**Affiliations:** 1 National Institute of Medical Welfare, Kangnam University, Yongin 16979, Korea; 2 Department of Biotechnology, The University of Suwon, Suwon 18323, Korea

**Keywords:** nanomedicine, vascular disease, VCAM-1, biomimetic, atherosclerosis, cancer

## Abstract

Nanotechnology has been developed to deliver cargos effectively to the vascular system. Nanomedicine is a novel and effective approach for targeted vascular disease treatment including atherosclerosis, coronary artery disease, strokes, peripheral arterial disease, and cancer. It has been well known for some time that vascular disease patients have a higher cancer risk than the general population. During atherogenesis, the endothelial cells are activated to increase the expression of adhesion molecules such as Intercellular Adhesion Molecule 1 (ICAM-1), Vascular cell adhesion protein 1 (VCAM-1), E-selectin, and P-selectin. This biological activation of endothelial cells gives a targetability clue for nanoparticle strategies. Nanoparticle formation has a passive targeting pathway due to the increased adhesion molecule expression on the cell surface as well as increased cell activation. In addition, the VCAM-1-targeting peptide has been widely used to target the inflamed endothelial cells. Biomimetic nanoparticles using platelet and leukocyte membrane fragment strategies have been promising techniques for targeted vascular disease treatment. Cyclodextrin, a natural oligosaccharide with a hydrophobic cavity, increase the solubility of cholesterol crystals at the atherosclerotic plaque site and has been used to deliver the hydrophobic drug statin as a therapeutic in a targeted manner. In summary, nanoparticles decorated with various targeting molecules will be an effective and promising strategy for targeted vascular disease treatment.

## 1. Introduction

**Figure 1:**
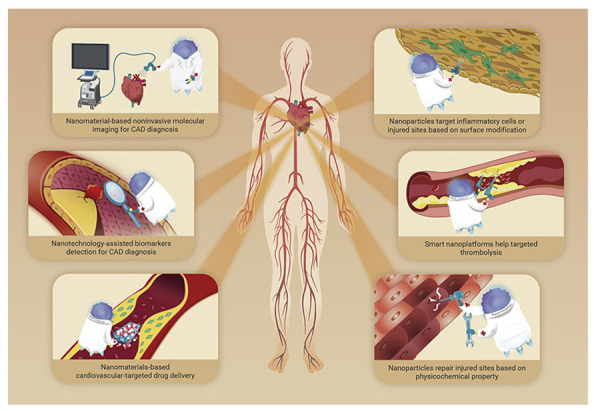
Feasible advances for the diagnosis and treatment of cardiovascular diseases (CVDs). Figure adapted with permission from Hu et al, 2022 [20].

Cardiovascular disease (CVD) refers to any disorder that affects the heart or blood vessels. CVD is frequently linked to fatty deposits in the arteries and an elevated risk of blood clots. Atherosclerosis, coronary artery disease (CADs), strokes, peripheral arterial disease, rheumatic heart disease, and other heart and blood vessel disorders are all classified as CVDs [1,2,3,4,5]. They have also been linked to arterial damage in organs such as the brain, heart, kidneys, and eyes [6,7,8,9]. According to the World Health Organization (WHO), CVDs are the leading cause of death worldwide, claiming the lives of 17.9 million people each year. Heart attacks and strokes account for more than four out of every five CVD deaths, with one-third of these deaths occurring before the age of 70 [10]. Current CVD treatment methods include management approaches such as exercise or dietary patterns, traditional medication treatment at the preventive level, and surgery, which places a heavy burden on the patient [11,12,13]. Even though the aforementioned treatments lessen symptoms and decrease mortality rates, both therapeutics have some limitations. Most CVDs are chronic due to tissue inflammation damage accumulated over a long period of time, and therefore, long-term drug administration for therapeutic purposes raises concerns about side effects such as liver failure, the weakening of renal function, and the malfunction of other organs [14,15,16]. In addition, these conventional drug formulations had inherent limitations including low water solubility, potential drug resistance, and poor biological efficiency [17,18,19]. Some patients who suffer from severe progressive vascular diseases need to consider surgical operations. Although significant contributions to reduce the symptoms and mortality of many vascular diseases have been made due to the excellent development of surgical techniques, surgery-related risks including inflammation, restenosis, and long recovery time are still a huge burden on patients. Therefore, establishing novel CVD therapeutics beyond conventional medication methods and surgical interventions are needed to increase the therapeutic effects and reduce the surgery-related risk burden on patients (Figure 1).

In the past decades, nanomedicine has been massively investigated for various disease models including cancer, CVDs, and diabetes [21,22,23,24,25]. Nanomedicine is the use of nanotechnology to accomplish disease treatment at the nanometer (~10^–9^ m)-size scale. Unlike conventional medicine, it makes the properties of materials suitable for use at the nanometric scale, which often differ from the same substance at a larger size in terms of physics, chemistry, and biology. Furthermore, the nanometric scale is also the size of many biological mechanisms in the human body, allowing nanoparticles (NPs) to potentially cross natural barriers to reach the new delivery sites and interact with DNA or small proteins at various levels, whether in blood or within organs, tissues, or cells. Nanomedicine currently covers a number of healthcare fields including targeted nanotherapeutics [26,27,28], medical imaging [29,30,31], diagnostics [32,33,34], vaccines [35,36], and regenerative medicines [37,38]. In particular, targeted nanotherapeutics can increase the therapeutic effects while reducing off-target side effects. Despite the development of various research using nanomedicine, nanomedicine for CVD purposes is still challenging. Because of the wide range of lesion areas of common vascular diseases, it is complicated to accurately deliver the therapeutic cargos to specific blood vessel lesions. Therefore, a targeted nanomedicine system that can accurately deliver a therapeutic drug to a desired location in a desired amount is expected as a promising treatment method for vascular disease. These approaches will be able to provide a basis for a platform to develop CVD therapeutics (Table 1).

In this review, we will discuss the recent progress of nanomedicine for targeted CVD treatment. Furthermore, current studies, trends, and future perspectives in targeted CVD treatment are also discussed.

## 2. Properties of NPs for Targeted Drug Delivery and Types of NPs Used for CVD

**Figure 2:**
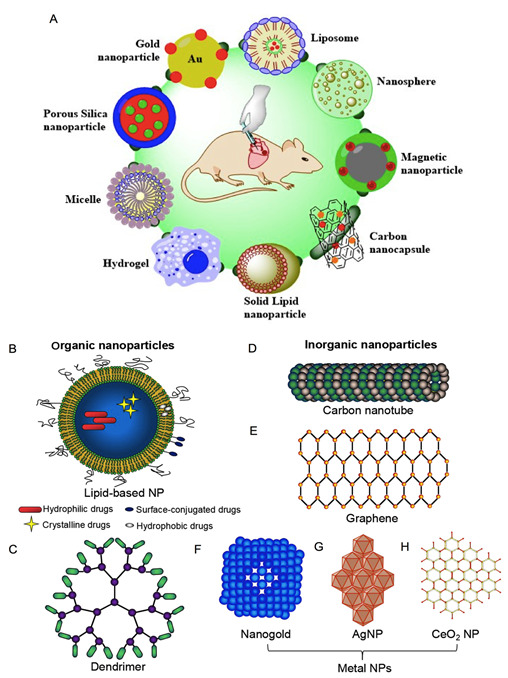
(**A**) Outline of the forms of drug delivery systems to treat heart disorders. Figure adapted with permission from Skourtis et al, 2020 [70]. Different NPs generally utilized in CVD therapeutic studies such as organic NPs (**B**,**C**), inorganic (**D**–**H**), and hybrid NPs. Panel reproduced with permission from Fan er al., 2020 [71].

A number of studies suggested that the physiochemical properties of NPs, including shape, size, composition, charge, and surface chemistry, would typically influence their interaction with biological systems, thus altering their in vivo dissemination [39]. Since NPs are composed of materials designed at the molecular level, they are generally small-scale nanospheres [40]. In this way, they are able to move more spontaneously in the human systems than to big-sized materials. Small-sized nanoscale particles demonstrate exclusive chemical, structural, magnetic, mechanical, and biological characteristics. Particle size and distribution are the main critical features of NPs. They regulate the in vivo distribution, biological consequence, toxicity, and targeting capability of these delivery systems. Moreover, they can affect drug loading and release, and the stability of synthesized NPs. Small-sized particles have a bigger surface area-to-volume ratio; thus, a high number of the drugs made up of smaller particles could lead to prompt drug release, unlike large-sized particles with big cores that permit more of the drug to be encapsulated in each particle, providing a gradual release [41]. Therefore, the regulation of particle size delivers a method of adjusting the drug-release frequency. Along with the particle size, the shape of NPs also affects the intracellular release of therapeutics [42]. Apart from this, the zeta potential of an NP is generally used to distinguish its surface charge property [43]. It reveals the electrical potential of NPs and is affected by the alignment of the NPs and the medium in which they are dispersed. It has been known that NPs with a zeta potential more than ± 30 mV are quite stable in suspension, because the surface charge counteracts their aggregation. Numerous main mechanical characteristics that define the material performance while loading include rigidity, elasticity, hardness strength, and stiffness [44]. Based on the earlier literature, NPs can be categorized as inorganic, organic, or hybrid (Figure 2). While inorganic NPs are favored in shaping diverse functions and characteristics, organic NPs often show better biocompatibility. In the case of organic–inorganic hybrid NPs, the organic functional groups merge the particular features of the inorganic counterparts to develop effective utility for different biological applications [45]. Currently, the application of hybrid NPs for gradual drug release has been attracting increasing interest, especially to enhance the selectivity and efficiency of the drugs through merging the features of inorganic and organic constituents into one single NP system. A nanocarrier is an NP used for the delivery of a cargo, such as a therapeutic particle. The variety of existing NPs for drug delivery is extensive and involves dendrimers, polymeric NPs, carbon nanotubes (CNTs), metallic NPs, or lipid-based structures, for instance liposomes or micelles.

### 2.1. Organic NPs

#### 2.1.1. Lipid-Based NPs

Generally, lipid-based NP preparations involve solid–lipid NPs, nano-emulsions, and lipid–drug conjugates; each largely consist of physiological lipid analogs with stabilizers such as surfactants [46]. Based on the size of the lipid-based NPs, they are designated as micelles (~10–15 nm), liposomes (~90–100 nm), or polymeric NPs [47,48]. Among these, polymeric NPs have received much attention in CVD nanomedicine owing to their changing nature and feasible reabsorption in the biological system for high efficiency. It is worth mentioning here that polymeric NPs, such as polyglycolic acid, polylactic acid, and polylactic-co-glycolic acid (PLGA), are FDA-permitted polymers. Between these polymers, PLGA has been broadly tested as a drug carrier for CVD treatment [49,50,51]. To date, there are no liposomal drug platforms accessible for the management of CVDs in human systems. However, liposomal alendronate platforms were found to be secure in early-phase clinical trials for infusion during the time of percutaneous coronary interference [52].

#### 2.1.2. Dendrimers

Dendrimers own the benefit of improving the binding space upon adjustment of their outer surface with a few antibodies or ligands for active targeting [53]; moreover, they are able to hold drugs with low solubility [54]. Xue et al. [55] disclosed that the apoptosis induction and infraction size in cardiomyocytes were remarkably suppressed after being given a single intravenous dose of dendrimer, conjugated with a microRNA-1 inhibitor as seen in the myocardial infarcted (MI) mouse system. Currently, NP distribution via intravenous injection with targeting peptides is emerging as a promising therapeutic approach. In agreement, Xue et al. [56] described an initial targeting treatment for MI mouse using the tail vein injection with anti-miR-1 antisense oligonucleotide-loaded myocardium-targeting dendrimer.

### 2.2. Inorganic NPs

#### 2.2.1. Carbon-Based NPs

CNTs is a well-suited drug carrier for improved diffusion in the cells and also for contributing privileged drug functions [57]. Their electrical, optical, and mechanical characteristics make them a favorable candidate for possible therapeutic applications [58]. Furthermore, some studies have verified the promising possibilities of CNTs in cardiac tissue engineering, for instance, to promote the growth and function of cardiomyocytes [59,60] and stimulate the formation of gap junctions [61,62]. Unlike these works, other examinations have proposed that scaffolds comprising CNT and col-hydrogel might be encouraging injectable biomaterial to distribute cells and drugs for tissue reconstruction in infarcted myocardial tissues [58,63].

#### 2.2.2. Metal NPs

Gold NPs are one of the broadly utilized nanocarriers for the distribution of cardioprotective drugs. The different physiochemical characteristics, for instance, the exceptional stability, greater safety, and robust biocompatibility of several gold NPs, makes them prospective candidates in nano biomedicine [64]. It has been generally acceptable and claimed that conjugated drugs are clinically highly efficient and accurate. Even Simdax has been declared a clinically safe and sanctioned drug for the therapeutics of heart disease. Additionally, Simdax conjugated on gold NPs displays cardioprotective outcomes in vivo rat models with heart failure [65]. In another report, gold NPs given via intravenous injection can also recover myocardial injury in rat models [66]. Comparable to the organic NPs, the intramyocardial injection of a nano-complex (graphene oxide conjugated with the VEGF-165 gene) in the acute MI rat model demonstrates remarkable decrease in infarct size and capillary density improvement [67]. Cardiac tissue regeneration is an approaching therapeutic technique for the handling of CVDs. In addition, laponite NPs loaded with extracellular matrix hydrogel improved the phenotypic expansion of cardiac proteins along with cardiac cell compatibility [68].

### 2.3. Organic–Inorganic NPs

As of now, significance in the applications of numerous organic–inorganic hybrid NPs has risen excessively. Hybrid NPs combine the characteristics of inorganic and organic building units and form NPs with enhanced physical and chemical attributes, for example, good particle diameter and surface charge [45]. Moreover, Zhu et al. [69] fabricated inorganic–organic hybrid silica NPs for gene transfection delivery in bone marrow-derived mesenchymal stem cells and consequent in vivo cardiac reconstruction.

## 3. Biological Markers in CVDs for Targeted Nanomedicine

The biological marker, also known as a biomarker, represents measurable and quantifiable biological parameters such as a specific enzyme concentration, phenotype distribution, and the expression of certain biological substances [72]. In particular, diagnosis through the discovery of the biomarkers of various CVDs contributes to early detections, risk predictions, novel clinical approaches, and finally decreases the mortality of CVD patients. The expression of these biomarkers is caused by pathophysiological changes that occur as the cardiovascular disease progresses, and these markers are involved in various processes such as inflammation, platelet activation, plaque instability, and systemic stress [73]. There are several biomarkers for the targeted nanomedicine treatment of vascular diseases.

**Figure 3:**
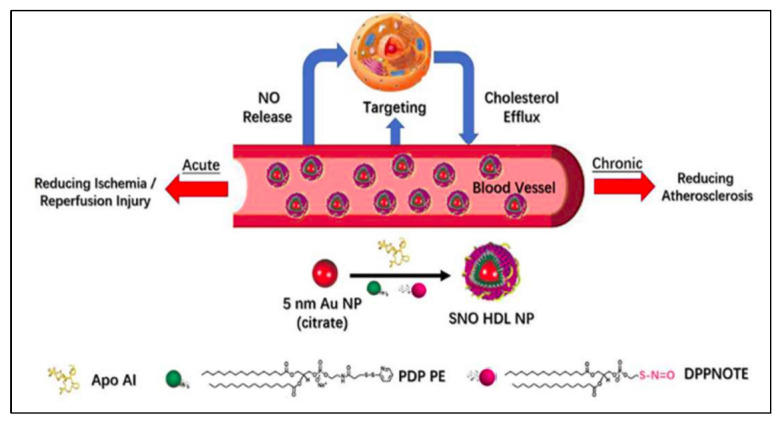
HDL-like NPs as a biomimetic nanotherapy for CVDs. Reprinted with permission from [78] Copyright 2018, American Chemical Society.

Cholesterol is transported throughout the body by lipoproteins. Lipoproteins can be distinguished from one another most simply by their density, which is directly correlated with the amount of either apolipoprotein A-I (ApoAI) or apolipoprotein B (ApoB). A higher risk of coronary heart disease is linked to high cholesterol levels in lipoproteins containing ApoB, specifically in VLDL (very low-density lipoprotein) and even more so in LDL (low-density lipoprotein) [74]. In numerous types of research assessing the concentration of ApoA-I, it has been demonstrated that ApoA-I-containing lipoproteins (usually HDL, high-density lipoprotein) are negatively linked with CHD [75]. Due to HDL cholesterol’s role in reverse cholesterol transport, which is the process by which peripherally deposited cholesterol is transported back to the liver for elimination, there is evidence that higher levels of HDL cholesterol may lessen the risk of cardiovascular disease [76,77]. In particular, HDLs that are natural targeted NPs of vascular diseases (Figure 3) and transport cholesterol in systemic circulation have been used as targeted nanomedicine and imaging agents [78,79,80,81]. These HDL-based NPs can be used as not only targeted delivery platforms to deliver therapeutic cargos and imaging agents of biomimetic nature, but also anti-inflammatory molecules given their characteristics for CVDs.

Endothelial cell (EC) dysfunction and activation after the penetration of LDL cholesterol induce the overexpression of adhesion molecules including intracellular cell adhesion molecule-1 (ICAM-1), vascular adhesion molecule-1 (VCAM-1), and endothelial leukocyte adhesion molecule-1 (ELAM-1, or selectin). The elevated level of circulating adhesion molecules predicts atherosclerosis and cardiovascular events and it is linked to cardiovascular risk factors [82,83,84]. These inflammation markers are another key biomarker for vascular disease for targeted nanomedicine. VCAM-1, an immunoglobulin superfamily glycoprotein, is expressed on the surface of the activated endothelium in the early stage of atherosclerosis [85,86]. In addition, atherosclerotic lesion development decreased in genetically modified mice with diminished VCAM-1 function [87,88]. Interestingly, MRI and optical imaging identified a linear peptide affinity ligand, VHPKQHR, a known ligand for VCAM-1-decorated NPs colocalized to VCAM-1-expressing ECs compared to non-targeting NPs [89]. This result denotes that VHPKQHR peptide can be a targeting molecule to VCAM-1-overexpressing ECs, which is the early biomarker for atherosclerosis.

## 4. Strategies for Targeted Nanomedicine for CVDs

**Figure 4:**
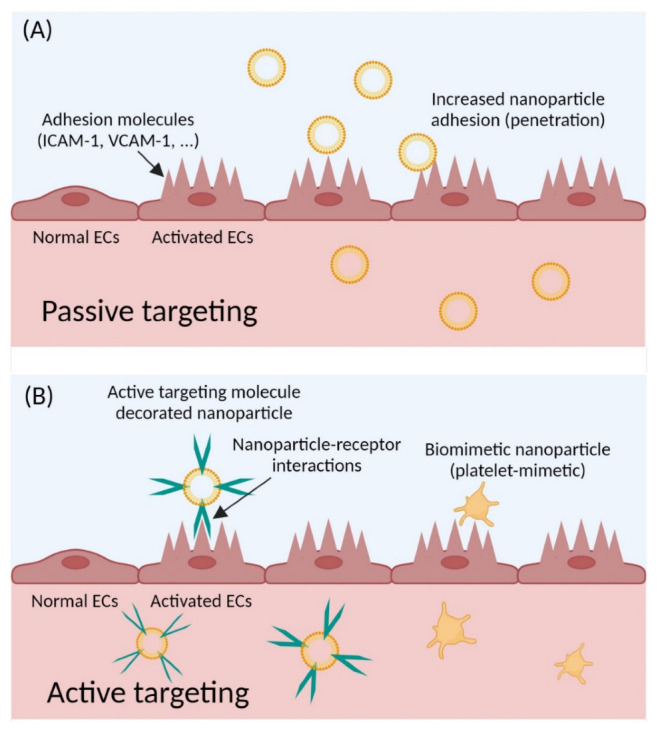
Targeted delivery of NPs. (**A**) Passive targeting is accomplished by increased nanoparticle adhesion to the adhesion molecules of activated endothelial cells, and (**B**) the active targeting method needs targeting molecule-decorated nanoparticle and biomimetic NPs.

A profound understanding is required of the mechanisms associated with disease progression and therapeutic targets and the drugs required to conquer physiological barriers, especially circulation to different organs, and then from tissue to cells, to reach therapeutic targets. A nanocarrier must overcome these physiological barriers for successive targeting in nanomedicine. Commonly, biological areas, for instance, the vascular endothelium or the passageway of NPs via cellular tissues, serve as barriers for the nano-sized materials. The smaller size of NPs permits them to go through the cellular membrane or other barriers, e.g., the blood–brain barrier, for the targeted drug delivery. Due to the steep rise in nanotechnology usage, the use of targeted delivery strategies has dramatically increased for treatment options against various diseases over the past few decades. There are two different targeting strategies that use NPs: passive targeting and active targeting. Passive targeting can be achieved by the enhanced permeability and retention (EPR) effect in cancer treatment [90,91]. The EPR effect represents a general pathophysiological mechanism, whereby non-targeted biological molecules or nano-sized particles can steadily accumulate in the tumor vascularized area due to increased vascular permeability, and thus achieve the passively targeted accumulation of anticancer compounds into inflamed regions and tumor tissues. Passive targeting allows for the efficient localization of NPs within lesion areas, whereas active targeting permits the active uptake of NPs by conjugating targeting molecules to nanoparticles. Either passive or active targeting methods are the best potent solution to increase NP accumulation in the desired area of the lesion in CVDs (Figure 4).

### 4.1. Passive Targeting

Atherosclerosis is a chronic inflammatory disease that starts with EC activation [92,93,94,95,96]. ECs in the arterial wall activated by chemical, mechanical, and immunological interactions increase the expression of adhesion molecules such as ICAM-1, VCAM-1, E-selectin, and P-selectin (Figure 4A) [97,98,99]. These molecules lead to monocyte adhesion and the differentiation of intimal macrophages. Activated ECs and increased adhesion molecules on ECs allow the preferential penetration of NPs into ECs via the passive targeting method. The critical physiological change of the inflamed region induced by activated ECs for passively targeted NP accumulation is increased vascular permeability [100]. Normal endothelial cells act as endothelial barriers, which can regulate endothelial junctions in the control of homeostasis. In the chronic inflammation of ECs, the normally tight junctions undergo a phenotype change into a leakier status. This phenotype change increases the passive accumulation of the macromolecules with a large molecular weight as well as the NPs through the EPR effect. Although this effect has not received much attention for diverse vascular diseases, a recent study revealed that cyclodextrin NPs have an increased accumulation in the atherosclerotic plaque lesions compared to non-targeting cyclodextrin molecules [101]. The NPs of cyclodextrin polymers of ~10 nm in diameter accumulated at the atherosclerotic plaque site due to their long circulation time and by evading the renal clearance system, whereas cyclodextrin solution was rapidly eliminated from the bloodstream. These findings support the conclusion that NP formations can passively target the lesion area due to the phenotype change of leaky junctions due to activated ECs and increased circulation time.

### 4.2. Active Targeting

As shown in Figure 4B, the active targeting strategy includes NP uptake by lesion area cells via NP receptor interactions. For the active targeting method, NPs need to be decorated with targeting molecules such as VCAM-1-targeting peptide or biomimetic surface proteins. In addition, recent research revealed that cyclodextrin can help to regress the atherosclerosis via macrophage reprogramming [102] and it can dissolve the cholesterol crystals in the plaque region [103]. These results indicate that cyclodextrin can be a promising targeted therapeutic approach against the development of atherosclerosis.

VCAM-1 is an immunoglobulin superfamily glycoprotein that is expressed on activated endothelial cells, macrophages, and smooth muscle cells (SMCs) and plays a role in the inflammation and development of atherosclerotic plaques [88,97,104]. Since the VHPK peptide (VHPKQHR) has high specificity and affinity to VCAM-1 on the endothelium in atherosclerotic plaque, the VCAM-1-targeting peptide, VHPK peptide, has been widely used for imaging and targeting the atherosclerosis lesion [89,105]. Kheirolomoom and his colleagues developed a VCAM-1-targeting peptide containing lipid NPs to target the atherosclerotic plaque on endothelial cells [106]. The VHPK peptide-bearing lipid NPs containing miR-712 as a therapeutic cargo had a particle size of 167 ± 40 nm and a round shape of single or bilayer membrane. TNFα (3 ng/mL)-pretreated mouse endothelial cells induced an increased VCAM-1 expression on the endothelial cell surface and the VHPK NPs was abundantly observed in the cytosol of the activated cells. These VHPK peptide-conjugated lipid NPs delivered anti-miR-712, which silences miR-712 levels known as pro-atherogenic mechanosensitive miRNAs to targeted inflamed endothelium in a mouse model, resulting the downregulation of plaque development. In addition, VHPKQHR peptide-incorporated NPs have been used for the non-invasive diagnosis of atherosclerosis via magnetic resonance imaging detection [107,108]. FITC (fluorescein isothiocyanate)-labeled VHPKQHR peptide-loaded Fe_3_O_4_@SiO_2_ NPs (FITC-VHP-Fe_3_O_4_@SiO_2_) were constructed for the MRI detection of atherosclerotic regions as an efficient targeted contrast agent. The NPs had good dispersion properties and a positively charged particle surface. The FITC-VHP-Fe_3_O_4_@SiO_2_ has low cytotoxicity against Raw264.7 (immune cells) and MAECs (mouse aortic endothelial cells, non-immune cells) as well as good blood compatibility. The NPs showed a clear increase of T2 image signal intensity on the atherosclerotic plaque region and the significant accumulation of the FITC-VHP-Fe_3_O_4_@SiO_2_ NPs in the dissected aortic vessel samples. These results suggested that VHPKQHR peptide conjugation can be an ideal option to target atherosclerotic plaque. VHPK-conjugated poly(β-amino ester) (pBAE) NPs were used to deliver anti-miR-712 to inflamed ECs. These NPs showed an increased therapeutic effect in an atherosclerotic plaque-developed mouse model, which was designed to induce atherosclerosis in the carotid via partial carotid ligation [109,110]. Overall, the conjugating of VCAM-1-targeting peptide (VHPK peptide) has been investigated as an efficient and promising targeting strategy against inflamed ECs, which is the region from which CVD originates.

Another active targeting method towards atherosclerosis plaque is using biomimetic particles. Rapamycin-loaded NPs, which have the leukocyte surface membrane decoration, leukosome, were developed and evaluated to reduce vascular inflammation [111]. Rapamycin is a promising anti-atherosclerotic agent and is an inhibitor of the mammalian target of the rapamycin (mTOR) pathway [112,113]. After the injection of rapamycin-loaded leukosome NPs, decreased proliferating macrophages in the aorta were reported as well as decreased MCP (monocyte chemoattractant protein)-1, IL (interleukin)-1b, and MMP (matrix metalloproteinases) activity in the aorta. Another study using leukocyte membrane-decorated NPs revealed that biomimetic NPs can target the atherosclerosis [114]. Leukocyte membrane fragments were used to prepare the magnetic nanoclusters and the anti-inflammatory drug simvastatin was embedded to the nanoparticle. These NPs showed excellent anti-atherosclerotic effects by decreasing the inflammation level and oxidative stress, and promoting cholesterol efflux.

Platelet membrane is also used to prepare biomimetic liposomes (platelet-mimetic hybrid liposomes) to make a direct interaction between the NPs and activated ECs [115]. During the progression of atherosclerosis, platelets play a critical role in atherosclerosis initiation to adhere to activated ECs. Platelet-mimetic hybrid liposomes showed a significant increase of plaque accumulation and plaque penetration. These results demonstrated that leukocyte and platelet membrane fragments can be a promising targeting strategy against activated ECs, which is the starting phenotype of various chronic CVDs.

As mentioned earlier, cyclodextrin-based NPs can be a promising targeting molecule for targeted CVD treatment. Cyclic oligosaccharide 2-hydroxypropyl-β-cyclodextrin [116] induces increased cholesterol crystal solubility, preventing atherosclerosis [102]. The cyclodextrin treatment against a murine atherosclerosis model reduced the plaque size of atherosclerosis and promoted plaque regression with a continuous high-cholesterol diet. In addition, a cyclodextrin polymer represented NP formation, which could accumulate at the plaque site [101], and due to the hydrophobic cavity of cyclodextrin, it was used as a hydrophobic drug carrier [103]. Simvastatin, which is widely used as a cholesterol-lowering drug to reduce the risk of CVDs, was loaded into the hydrophobic cavity of cyclodextrin. After lipid coating and homogenization, cargo-switching NPs were prepared, and these NPs showed a cargo-switching ability from loaded statins to cholesterol crystals at the atherosclerotic plaque site. This platform increased the targeted therapeutic effect at the plaque site due to the selective statin release mechanism, and these findings demonstrated that cyclodextrin can be a promising atherosclerotic plaque-targeting material.

## 5. Challenges and Future Perspectives

Although there are a number of promising results from nanotechnology-based CVD targeted therapy, critical roadblocks still remain in the effective treatment of CVDs, including low efficacy, decreased circulation time, and off-target effects from the delivered NPs. In this review, we have described the current research of targeted nanomedicine for CVD treatment. In the passive targeting method, the nano-sized particles demonstrate strong advantages compared to water-soluble molecules. In addition, more options were discussed for the active targeting method, including VCAM-1-targeting peptides, biomimetic membrane fragment hybridized NPs and liposomes, and cyclodextrin NPs. Despite the development of these targeting strategies against vascular disease, there are still challenging steps for future directions. First, a novel biomarker for vascular disease should be investigated. During the chronic development of vascular disease, extensive genotype and phenotype changes were observed over a long period of time. Therefore, the discovery of new biomarkers may provide an opportunity to find various target substances in targeted nanomedicine-based vascular disease therapy. Second, it is necessary to develop a range of peptide sequences that can target various biomarkers. Currently, the VCAM-1-targeting peptide, VHPKQHR, is the most successful targeting peptide against the early stages of atherosclerosis plaque development. Numerous studies using VCAM-1-targeting peptides showed excellent results in targeting the plaque regions, from non-invasive diagnosis imaging platforms to targeted therapeutic cargo delivery. Likewise, novel peptide sequences to target various biomarkers from the early stage to the severe status of pathology have to be investigated. Third, a new targeting strategy using various types of NPs should be established. As mentioned, current conventional medication methods have their own limitations such as low water solubility, potential drug resistance, and poor biological efficiency; therefore, the aggressive usage of nanotechnology is needed for vascular disease. Moreover, when combining NP strategies, various administrative routes should be devised. The majority of NP treatments were administered via intravenous injection. Nobody wants to carry out intravenous injections for lowering the risk of CVD. In particular, when it comes to oral delivery, NP oral delivery must be improved in terms of their bioavailability during the digestive process. Lastly, personalized medicine techniques must be improved. Due to the chronic inflammation characteristics of various CVDs, the vascular disease treatment usually takes a long time, and it must be safe and stable enough to be used repeatedly. In the near future, improvements in nanomedicine, personalized therapy, and material science are projected to deliver significant functional improvements and EC restoration in patients with vascular diseases. Exploring the relationship among patient systems, disease heterogeneity, and nanomedicine could help conquer the biological obstacles of nanotheranostics. Prior to conducting clinical phase trials, nanoformulations should be intensively certified in preclinical in vivo model systems. Various natural products derived from active drugs and nanodrugs with cardioprotective actions are awaiting future investigation and clinical translations.

## Figures and Tables

**Table 1:** List of nanoplatforms for target delivery of various drugs to the sites of cardiovascular diseases (CVDs). Table reproduced from Hu et al., 2022 [20].

Drug Type	Loaded Drug	Nanoplatforms	Disorders	Mechanism of Action	Surface Modifications	Model of Use/Animal	Administration Route
Statins	atorvastatin atorvastatin	HA-ATV-NP Oxi-COS/MM-AT-nps	atherosclerosis atherosclerosis	suppression of inflammation suppression of inflammation	hyaluronan proteins derived from macrophages membrane	in vitro; in vivo, ApoE-/- mice in vitro; in vivo, ApoE-/- mice	intravenous injection intravenous injection
Rapamycin	rapamycin rapamycin	PFN1-CD-mnps liposome	atherosclerosis atherosclerosis	suppression of inflammation suppression of inflammation	profilin-1 antibody membrane protein from leukocytes	in vitro; in vivo, ApoE-/- mice in vitro; in vivo, ApoE-/- mice	intravenous injection retro-orbital injection
Traditional Chinese medicine	Sal B, PNS	RGD-S/P-lpns	AMI		RGD peptide ligand	in vivo, SD rats receiving experimental MI	intravenous injection
Small molecule agonists/inhibitors	SMI 6877002	rHDL NPs	atherosclerosis	inhibition of monocyte recruitment; suppression of plaque inflammation	ApoA-I	in vitro; in vivo, ApoE-/- mice, cynomolgus monkeys	intravenous injection
Small molecule agonists/inhibitors	SNO	SNO-HDL NPs	atherosclerosis		ApoA-I	in vitro; in vivo, ApoE-/- mice	intravenous injection
siRNA	siCamk2g	G0-C14 PLGA NPs	atherosclerosis	promotion of efferocytosis	S2P peptide (CRTLTVRKC)	in vitro; in vivo, Ldlr-/- mice	intravenous injection
miRNA	miR-145	PAM	atherosclerosis	promotion of the contractile VSMC phenotype	MCP1/CCL2	in vitro; in vivo, ApoE-/- mice	intravenous injection
miRNA switches	miRNA switches	mRNA-p5RHH nanoparticle	restenosis	specific inhibition of the VSMCs and inflammatory cells		in vitro; in vivo, C57BL6/J mice undergoing femoral artery wire injury	intravenous injection

## Data Availability

Not applicable.
